# Comparing genomic signatures of domestication in two Atlantic salmon (*Salmo salar* L.) populations with different geographical origins

**DOI:** 10.1111/eva.12689

**Published:** 2018-12-07

**Authors:** Maria E. López, Laura Benestan, Jean‐Sebastien Moore, Charles Perrier, John Gilbey, Alex Di Genova, Alejandro Maass, Diego Diaz, Jean‐Paul Lhorente, Katharina Correa, Roberto Neira, Louis Bernatchez, José M. Yáñez

**Affiliations:** ^1^ Facultad de Ciencias Veterinarias y Pecuarias Universidad de Chile Santiago Chile; ^2^ Facultad de Ciencias Agronómicas Universidad de Chile Santiago Chile; ^3^ IBIS Institut de Biologie Intégrative et des Systèmes Université Laval Québec City Québec Canada; ^4^ Centre d’Écologie Fonctionnelle et Évolutive Unité Mixte de Recherche CNRS 5175 Montpellier France; ^5^ Marine Scotland Science Freshwater Fisheries Laboratory Faskally Pitlochry UK; ^6^ Laboratory of Bioinformatics and Mathematics of the Genome Center for Mathematical Modeling (UMI 2807 CNRS) and Center for Genome Regulation (Fondap 15090007) Universidad de Chile Santiago Chile; ^7^ Aquainnovo Puerto Montt Chile; ^8^ Núcleo Milenio INVASAL Concepción Chile

**Keywords:** *Salmo salar*, selective sweeps, single nucleotide polymorphisms

## Abstract

Selective breeding and genetic improvement have left detectable signatures on the genomes of domestic species. The elucidation of such signatures is fundamental for detecting genomic regions of biological relevance to domestication and improving management practices. In aquaculture, domestication was carried out independently in different locations worldwide, which provides opportunities to study the parallel effects of domestication on the genome of individuals that have been selected for similar traits. In this study, we aimed to detect potential genomic signatures of domestication in two independent pairs of wild/domesticated Atlantic salmon populations of Canadian and Scottish origins, respectively. Putative genomic regions under divergent selection were investigated using a 200K SNP array by combining three different statistical methods based either on allele frequencies (LFMM, Bayescan) or haplotype differentiation (Rsb). We identified 337 and 270 SNPs potentially under divergent selection in wild and hatchery populations of Canadian and Scottish origins, respectively. We observed little overlap between results obtained from different statistical methods, highlighting the need to test complementary approaches for detecting a broad range of genomic footprints of selection. The vast majority of the outliers detected were population‐specific but we found four candidate genes that were shared between the populations. We propose that these candidate genes may play a role in the parallel process of domestication. Overall, our results suggest that genetic drift may have override the effect of artificial selection and/or point toward a different genetic basis underlying the expression of similar traits in different domesticated strains. Finally, it is likely that domestication may predominantly target polygenic traits (e.g., growth) such that its genomic impact might be more difficult to detect with methods assuming selective sweeps.

## 1 INTRODUCTION

Domestication, “the process by which captive animals adapted to man and the environment he provides,” has led to several genetic changes over generations in various animal and plant species (Price, 1984). This evolutionary process may induce similar phenotypic changes in populations of independent origins within the same species when the same phenotypic traits are subjected to similar selective pressures. Phenotypes of these animals have progressively evolved due to the combined influence of domestication selection through reproduction in captivity and human directional selective breeding (Andersson, 2012). This may result in a remarkable phenotypic diversity within domestic species as well as a wide variety of genetic adaptations to both environmental conditions and production systems (Andersson, 2012, 2013). Domestication has thus shaped the genetic diversity of these species throughout history, and their present genomes may contain traceable signatures of selection (Utsunomiya, Pérez, O'Brien, Sonstegard, & Garcia, 2015).

In most fishes, domestication is a recent process compared with any other livestock species (Gjedrem, 2005; López, Neira, & Yáñez, 2015). Therefore, important knowledge gaps still remain about the consequences of domestication on behavior, physiology, and morphology of fishes (Teletchea & Fontaine, 2014) compared to domesticated mammals and birds, for which more data are available (Kelley, Brown, Therkildsen, & Foote, 2016; Lorenzen, Beveridge, & Mangel, 2012). Nevertheless, there is no reason to consider domestication of terrestrial and aquatic animals distinctively (Balon, 2004; Teletchea & Fontaine, 2014). Hence, we might expect that, similar to birds and mammals, selection for the phenotypes contributing to domestication goals has also impacted the extent and distribution of variability within the genomes of fishes. Indeed, domestication has greatly impacted the phenotypes of domesticated aquatic species (e.g., Atlantic salmon [*Salmo salar* L.], rainbow trout [*Oncorhynchus mykiss* W.], tilapia [*Oreochromis niloticus* L.], common carp [*Cyprinus carpio* L.]) in which selective breeding programs have been implemented, focusing on specific economic objectives (Gjedrem, 2012; Rye, Gjerde, & Gjedrem, 2010).

Detecting genomic signatures of selection is a major goal of modern population genetics (Fariello et al., 2014; Nielsen, Hellmann, Hubisz, Bustamante, & Clark, 2007) as it enhances our knowledge of the molecular mechanisms shaping the genome as well as providing functional information on specific genes/genomic regions that would be of interest for breeding programs (Qanbari & Simianer, 2014). Recently, the progress of high‐throughput and cost‐effective genotyping techniques has offered a unique opportunity to analyze large datasets of domesticated species to study genome changes in response to domestication events (Druet, Pérez‐Pardal, Charlier, & Gautier, 2013; Ma et al., 2015). In livestock species, genomewide analyses have already shown promising results in mapping traits of economic interest, such as genomic regions related to milk production in cattle (*Bos tourus*) (Qanbari et al., 2011), muscle development in pig (*Sus scrofa*) (Amaral et al., 2011), coat pigmentation and skeletal morphology in sheep (*Ovis aries*) (Kijas et al., 2012), or gait and size in horses (*Equus caballus*) (Petersen et al., 2013). The practical aspect of these population genomics studies lies on the possibility of detecting selected genes associated with traits of economic interest and acts as complement to gene mapping approaches (e.g., genomewide association studies [GWAS]) that may help to further genetically improve these traits on domestic species (Qanbari & Simianer, 2014). Furthermore, this knowledge is important from an evolutionary perspective by highlighting traits that have been exposed to natural and artificial selection as well as using this information to design and/or update breeding programs for conservation purposes worldwide (Brito et al., 2017; Cesconeto et al., 2017; Zhao, McParland, Kearney, Lixin, & Berry, 2015).

Recently, methods such as whole genome sequencing or genomewide SNP arrays have enabled the screening of a large part of the genome to detect signatures of selection in domestic and natural populations (Druet et al., 2013; Fuentes‐Pardo & Ruzzante, 2017; Ma et al., 2015). Moreover, new genome scan approaches have been developed with the goal of efficiently and accurately identifying genomic footprints of selection out of the thousands of markers screened (Jensen, Foll, & Bernatchez, 2016). Several analytical methods are now available, ranging from population differentiation analyses, based on *F*_ST_ calculation (Porto‐Neto, Lee, Lee, & Gondro, 2013), to environmental association methods (Cesconeto et al., 2017; Frichot, Schoville, Bouchard, & François, 2013), which aim to detect genetic variants associated with specific environmental factors. Population differentiation methods are expected to detect only strong signatures of selection (e.g., hard sweep), whereas alternative methods such as environmental association would be able to detect subtle signal of selection (e.g., soft sweep), which makes the combination of the two a particularly promising tool for uncovering any genomic footprints of selection (Benestan et al., 2016; Rellstab, Gugerli, Eckert, Hancock, & Holderegger, 2015).

Atlantic salmon aquaculture has a high economic value, and breeding programs have been implemented worldwide to improve genetic performance of domesticated individuals (Gjedrem, Robinson, & Rye, 2012; Rye et al., 2010). In this perspective, several studies have already detected evidences for genomic signatures of domestication in Atlantic salmon (Bourret, O'Reilly, Carr, Berg, & Bernatchez, 2011; Mäkinen, Vasemägi, McGinnity, Cross, & Primmer, 2014; Martinez et al., 2013; Roberge, Einum, Guderley, & Bernatchez, 2006; Vasemägi et al., 2012) and showed that these signatures were related to important biological functions including immunity, lipid transport, fatty acids, steroid metabolism, and cellular cycle/growth (Devlin, Sakhrani, Tymchuk, Rise, & Goh, 2009; Sauvage et al., 2010; Wringe et al., 2010). Yet, little is known about how the process of domestication altered the ancestral wild Atlantic salmon genome, partly due to the fact that the demographic history of wild Atlantic salmon populations is complex. Indeed, wild populations of Atlantic salmon exhibit complex patterns of hierarchical genetic differentiation at continental, regional, and local scales (Dillane et al., 2008; Dionne, Caron, Dodson, & Bernatchez, 2008; Moore et al., 2014; Perrier, Guyomard, Bagliniere, & Evanno, 2011; Spidle et al., 2003; Verspoor et al., 2005). These complex patterns are the product of postglacial colonization (King, 2007; Rougemont & Bernatchez, 2018) combined with contemporary ecological processes such as homing behavior, which maintains genetic differentiation among rivers, as well as spatially varying environmental conditions (Bourret et al., 2013; Moore et al., 2014; Rougemont & Bernatchez, 2018).

Across the entire Atlantic salmon natural range, European and North American populations exhibit a deep genetic divergence and belong to two divergent glacial lineages which likely separated more than 1,000,000 years ago (Rougemont & Bernatchez, 2018). While these two lineages did not share the same demographic history, domestic populations derived from them have been selected for similar economically important phenotypic traits such as growth, age at sexual maturity, disease resistance, and flesh quality (Gjedrem, 2012), which suggests that domestication may involve parallel evolution. Finding parallel genetic differences between these two populations would reinforce the hypothesis that candidate genomic regions may be linked to molecular processes underlying domestication. Indeed, signals of selection detected among farmed fish belonging to different origins are likely to result from selection rather than having been inherited by chance or from migration of selected allele across breeding strains, which limits false‐positive detection. In Atlantic salmon, previous transcriptomic studies showed that European and North American populations exhibit parallel differential gene expression profiles between hatchery strains and wild populations in the same set of genes (Roberge et al., 2006; Sauvage et al., 2010). Yet, Vasemägi et al. (2012) using 261 SNPs and 70 microsatellites loci found little support for parallel evolution as they found none of the candidate genomic regions potentially associated with domesticated and improved traits overlapped across three different lineages (Ireland, Sweden, and Canada). Using a genomewide analysis with a 6.5K SNP array, Mäkinen et al. (2014) revealed that candidate loci associated with domestication were located on different chromosomes for the North American and European lineages. In this study, we aimed at further investigating the genomic footprints of domestication as well as parallel genomic changes between two pairs of wild/domestic populations of Atlantic salmon with European and North American origins using a 200K SNP chip that may offer higher resolution than achieved in previous studies. We applied three complementary statistical approaches to identify genomic regions putatively under selection: (a) a genome scan based on population differentiation indices (*F*_ST_) (BAYESCAN), (b) genomewide environmental association (LFMM), and (c) haplotype extended patterns (Rsb). From the set of candidate genes detected by these approaches, we identified their biological function to delineate their potential importance in the process of domestication of Atlantic salmon populations.

## 2 MATERIALS AND METHODS

### 2.1 Studied populations

This study was conducted using two wild/domestic population pairs of Atlantic salmon from two different geographical origins: Canada and Scotland, for a total of 183 individuals (see Table 1 for details). The purpose of this design was to perform two independent comparisons between domestic strains and their wild counterpart populations. Samples from Canadian domestic population (Can‐D) were collected from a domestic strain of Atlantic salmon cultured in Chile. This strain was established from eggs obtained from the Gaspé Bay (where the Saint‐Jean River flows), Québec, in the 1950s (Withler, Supernault, & Miller, 2005). Using 11 microsatellites Withler et al. (2005) showed no evidence of introgression from other strains. We presume that fish of this strain were brought to the United States in the late 80s and early 90s and kept in a hatchery located in the state of Washington for about two generations before being introduced to Chile between 1996 and 1998 (J. P. Lhorente, personal communication). This strain has been subjected to intensive selection for rapid growth rate as the main objective of breeding, and also for low incidence of early sexual maturation (J. P. Lhorente, personal communication). This Chilean farmed population has been reared in the environmental conditions of the Los Lagos Region (42°S 72°O, Chile) for about four generations. The wild population from Canada (Can‐W) included adult individuals sampled through angling in Saint‐Jean River on the same year (Dionne et al., 2008). The Saint‐Jean River flows into Gaspe Bay, along with York and Dartmouth rivers, and salmon population from Saint‐Jean River is the largest one of the three. Furthermore, it has been shown that genetic differentiation among individuals from these three rivers is weak (θ_ST_ (Weir & Cockerham, 1984) = 0.011) (Dionne et al., 2008), enough to consider our samples as a good representation of the possible wild ancestors of domestic Canadian population used in this study. On the other hand, the Scottish farmed population (Sct‐D) is a strain cultivated in Chile and it was established with fish from Loch Lochy, located on the West Coast of Scotland. This strain was maintained in a commercial genetic improvement program based in Scotland (Johnston, Alderson, Sandham, Mitchell, et al., 2000) and is characterized by a high proportion of early maturing fish and faster growth rate than strains with high proportion of late maturing fish (Johnston, Alderson, Sandham, Dingwall, et al., 2000; Johnston, Alderson, Sandham, Mitchell, et al., 2000). In 1986, this Scottish farmed population was transferred to Los Lagos Region (42°S 72°O, Chile), presenting a high incidence of fish with early sexual maturity and rapid growth as well, based on field records from 2004. Since then, this population has been adapted to environmental, geographical, and climatic conditions in Chile and selected for rapid growth rate using mass selection for at least six generations (C. Soto, personal communication). The wild population from Scotland (Sct‐W) comprised juvenile individuals from South Esk River on the East Coast of Scotland collected in 2011, which is a region with sparse aquaculture activity; therefore, there is a low probability that escaped farmed fish had affected the genetic diversity of wild individuals. We used samples from this location, instead of fish from the West Coast of Scotland, as the latter has an important aquaculture activity and has also been stocked from different sources. Therefore, wild fish from this region does not represent a pure Scottish wild Atlantic salmon. Individuals were collected using electrofishing followed by anesthesia and partial fin‐clipping. All fish were released back to the capture location (Yáñez et al., 2016). Fish collecting work has been reviewed both by Marine Scotland Review Committee and the United Kingdom Home Office (Project License 60/4251).

**Table 1:** Atlantic salmon populations analyzed in this study with geographical origin and sample size (*n*)

Population code	Geographical origin	*n* a	*n*_QC_ b	%^c^	Observations
Can‐W	Canada	46	44	95.7	Wild population from Saint‐Jean River, Québec, Canada
Can‐D	Canada	40	37	92.5	Farmed population selected for fast growth rate and characterized for late sexual maturity
Sct‐W	Scotland	47	41	87.2	Wild population from the East Coast of Scotland.
Sct‐D	Scotland	50	43	86	Farmed population selected for fast growth rate and characterized for early sexual maturity

^a^Initial number of individuals. ^b^Number of individuals passing sample call rate of 90%. ^c^Percentage of individuals passing sample call rate of 90%.

### 2.2 Genotyping and quality control

Genomic DNA was extracted from fin clips using a DNeasy Blood & Tissue Kit (QIAGEN). For genotyping, we used an Affymetrix Axiom^®^ myDesign Custom Array of 200K SNPs, which was developed using thirteen European fish (Scottish and Norwegian origin) and seven North American fish, as described by Yáñez et al. (2016). SNP quality control (QC) was carried out using Axiom Genotyping Console (GTC, Affymetrix) and SNPolisher (an R package developed by Affymetrix), based on SNP clustering metrics and call rate status. Thus, the QC was performed by (a) removing SNP that did not match with high quality clustering patterns, which are defined according to the best practices recommended by Affymetrix through using SNPs with (i) PolyHighResolution (good cluster resolutions for homozygote and heterozygote samples and at least two occurrences of minor allele) and (ii) NoMinorHom (two distinctive cluster with no‐minor homozygous genotypes), (b) removing SNP loci with call rate lower than 0.95, and (c) discarding individuals with call rate lower than 0.90. Further filtering steps included removing loci deviating from Hardy–Weinberg equilibrium calculated for each population separately, and subsequently, we removed SNPs shared among all populations, and this stage was part of the validation of the SNP chip as described by Yáñez et al. (2016). Loci with minor allele frequency (MAF) lower than 5% within each pair of populations joined (i.e., Can‐W and Can‐D comprised one group; Sct‐W and Sct‐D comprised another group) using PLINK v1.07 (Purcell et al., 2007).

### 2.3 Basic statistics and structure analysis

To investigate population differentiation, we calculated the pairwise Weir and Cockerham's *F*_ST_ (1984) estimator across all loci among populations, using VCFTools (Danecek et al., 2011). Observed and expected heterozygosity (*H*_O_ and *H*_E_) were estimated using PLINK v1.07 (Purcell et al., 2007), and confidence intervals for these statistics were calculated using the R package *boot* with 1,000 bootstrap replicates (Canty & Ripley, 2016). Genomic distribution of *F*_ST_ was further analyzed by a kernel‐smoothing approach using the R package *Lokern*. A local bandwidth of ~400 was used for fitting the kernel‐smoothed regression line. Two clustering methods were used to assess the extent of genetic structure among the populations. First, we performed a principal component analysis (PCA) implemented in the R package *adegenet* (Jombart, 2008). Second, we used the maximum likelihood estimation of individual ancestries through ADMIXTURE (Alexander, Novembre, & Lange, 2009) software. ADMIXTURE analysis was run using 2,000 bootstraps, and the number of ancestral populations was set from 1 to 10 (*K*). The optimal *K* was selected based on the lowest cross‐validation error and a visual inspection of the co‐ancestry values for each individual.

### 2.4 Identification of selection signatures

Three methods were implemented to detect signatures of selection: (a) population differentiation, (b) environmental association, and (c) haplotype extended patterns. First, we used the population differentiation approach with a Bayesian likelihood method, implemented in BAYESCAN v.2.1, which identifies candidate loci under selection using differences in allele frequencies between populations. The algorithm uses a reversible‐jump Markov Chain Monte Carlo to explore models with or without selection. To estimate the probability that a locus is under selection, the program uses a Bayes factor for two models: one assuming selection and another assuming neutrality given the data (Foll & Gaggiotti, 2008). Bayescan was run using a pairwise differentiation test (i.e., wild vs. domestic strains). For both independent runs on Canadian and Scottish pair of populations, we used BAYESCAN v.2.1 default parameters. A Bayes factor between 10 and 32 (log_10_ = 1–1.5) for one locus is considered putatively under divergent selection, while between 32 and 100 (log_10_ = 1.5–2) and Bayes factors above 100 (log_10_ > 2) are considered putatively under very strong and decisive divergent selection, respectively. We used a Bayes factor of 10 (log_10_ = 1.30) as threshold to select candidate loci under selection.

Second, we performed an environmental association approach using latent factor mixed models implemented in the software LFMM (Frichot et al., 2013). This method calculates the correlations between allele frequencies and environmental variables. We defined *Domesticated* and *Wild* conditions as an environmental dichotomous variable (0 = wild; 1 = domesticated). This method can efficiently estimate random effects due to population history and isolation‐by‐distance and can reduce false‐positive associations compared to genome scans (Frichot et al., 2013). We applied the “*latent factor mixed models*” algorithm and calculated the |*z*| scores for all of the SNPs in both independent comparisons for Canadian and Scottish populations, using 20,000 iterations, 10,000 burn‐in iterations in the Gibbs Sampling algorithm and a seed of 1,000. The number of latent factors, which is the best number of clusters describing population structure of the original data, was chosen according to the results of the clustering analyses (i.e., PCA and ADMIXTURE) and as recommended by Frichot et al. (2013).

Finally, we investigated haplotype extended patterns using the *Standardized log‐ratio of integrated EHHS (iES) between pairs of populations* (Rsb) analysis (Tang, Thornton, & Stoneking, 2007). This test is based on the extended haplotype homozygosity (EHH) statistic and contrasts EHH patterns of the same haplotype between populations. The SNPs position on the genome is needed estimating EHH, which was possible thanks to the availability of the chromosomal‐level genome assembly for Atlantic salmon (Lien et al., 2016). Rsb is defined as the natural log of the ratio between iES_POP1_ and iES_POP2_, where iES is the integrated EHHS (site‐specific EHH) for each SNP within each population. As the Rsb values are normally distributed, a *Z*‐test was applied to identify significant SNPs under selection between wild and domestic strains of both Canadian and Scottish origins. A positive value of Rsb indicate iES_POP1_ is larger than iES_POP2_; therefore, Pop1 has longer haplotypes than Pop2, and hence, positive Rsb scores suggest that selection occurred in the alternative population (domestic population), whereas the negative scores suggest that selection occurred in the reference population (wild population). One‐sided *p*‐values were obtained as −log_10_(1‐Φ(Rsb)), where Φ(Rsb) represents the Gaussian distribution function for the Rsb statistic. We used −log_10_(*p*‐value) = 3 as a threshold to define significant Rsb values *Standardized log‐ratio of integrated EHHS (iES) between pairs of populations* using *rehh* R package (Gautier & Vitalis, 2012).

Bayescan and LFMM methods can directly handle the SNP genotypes, whereas Rsb requires haplotypes. We imputed the missing genotypes and inferred haplotypes from genotypes using BEAGLE v.3 (Browning & Browning, 2009) with default parameters. Phasing was carried out separately for each population; the SNP position on chromosome was obtained from the Atlantic salmon genome reference ICSAG_v2 (Lien et al., 2016).

### 2.5 SNP annotation and functional enrichment analysis

Genomic regions harboring each locus putatively under selection, detected by at least one method (BAYESCAN, LFMM or Rsb), were interrogated for genes annotated to the Atlantic salmon genome reference ICSAG_v2 (GenBank: GCA_000233375.4) using SnpEff v4.3 (Cingolani et al., 2012). This functional annotation is a complete representation of the genome containing 37,206 high‐confidence protein‐coding genes that have been assigned to a putative functional annotation based on homology within the SwissProt database (Lien et al., 2016).

Using the salmon transcripts, we performed a blastx on the zebrafish (*Danio rerio*) peptide reference database (downloaded from http://www.ensembl.org). For each unique peptide in this species, the corresponding gene id and associated gene name were retrieved from ensembl biomart database (retrieved from http://www.ensembl.org/biomart). Enrichment analysis was conducted using the online tool Panther Classification System (http://www.pantherdb.org) with default parameters.

## 3 RESULTS

### 3.1 Genotyping and SNP filtering

After genotyping QC, following the conditions recommended by Affymetrix described in Materials and Methods, 159,099 SNPs were categorized in two good quality metrics (a) poly high resolution (good cluster resolution of homozygote and heterozygote samples and at least two occurrences of minor allele) and (b) no‐minor allele homozygous (good cluster resolution with no‐minor homozygous genotypes). Thus, a total of 151,509 SNPs were anchored to a unique location on the last version of Atlantic salmon genome assembly (GenBank: GCA_000233375.4) (Yáñez et al., 2016). We removed 149 markers deviating from Hardy–Weinberg equilibrium. After filtering for minor allele frequency, we obtained 61,199 loci with MAF > 5% for both Canadian populations joined (i.e., Can‐W and Can‐D), and 130,586 loci with MAF > 5% for Scottish populations joined (i.e., Sct‐W and Sct‐D). The 55,406 common markers among four populations that passed MAF filtering were used for the following analyses. We also discarded individuals with call rate less than 90%, and 165 individuals were retained for further analyses. The detailed number of samples for each population is shown in Table 1.

### 3.2 Population differentiation and structure analyses

Using the set of markers with MAF > 5% common to all four populations revealed that measures of observed heterozygosity (*H*_O_) and expected heterozygosity (*H*_E_) were higher in Scottish than Canadian stocks. Overall, *H*_O_ was slightly higher than *H*_E_ in every population evaluated and heterozygosity levels were higher in both wild populations in comparison with their domesticated counterpart. Indeed, *H*_O_ was 0.404 (CI = 0.400–0.407) versus 0.353 (CI = 0.350–0.356) and 0.297 (CI = 0.294–0.299) versus 0.266 (CI = 0.264–0.269) in wild versus domesticated Scottish and Canadian populations, respectively. A similar trend was observed for *H*_E_ with wild Scottish exhibited the highest level (*H*_E_ = 0.397, CI = 0.394–0.400), followed by domestic Scottish (*H*_E_ = 0.343, CI = 0.340–0.346), while wild and domestic Canadian *H*_E_ was lower (*H*_E_ = 0.294, CI = 0.292: 0.297 and 0.244, CI = 0.242–0.247 respectively). All these genetic diversity measures were statistically significant from each other (*p* < 0.05, Kruskal–Wallis test).

The highest level of differentiation was found between both domestic populations originating from Canada and Scotland (*F*_ST_ = 0.393, CI = 0.390–0.396), although the *F*_ST_ between both wild populations was 0.286 (CI = 0.284–0.288). When combining wild and domestic individuals (of each geographical origin), Canadian and Scottish populations still showed a high level of genetic differentiation (*F*_ST_ = 0.293, CI = 0.290–0.295). A lower *F*_ST_ was observed between Canadian wild and domestic populations (*F*_ST_ = 0.171, CI = 0.169–0.173) which was twice as large as the *F*_ST_ estimated between wild and domestic Scottish populations (*F*_ST_ = 0.080, CI = 0.078–0.081).

**Figure 1:**
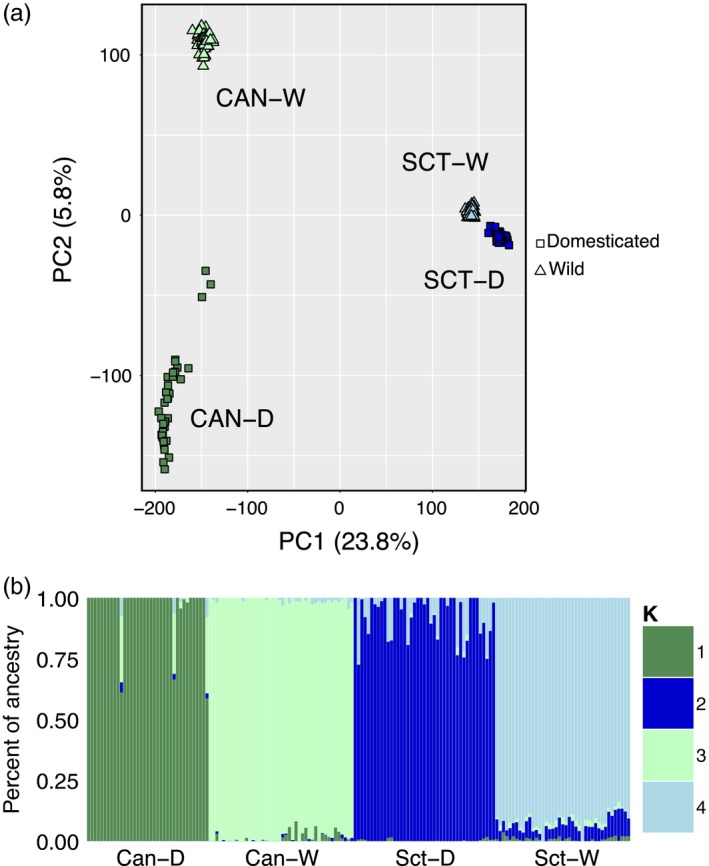
Population genetic structure. (a) Principal components analysis (PCA) of genetic differentiation among the 165 individuals, based on 55,406 polymorphic markers. Each point represents one individual, and different colors represent populations: Canadian domestic = dark green, Canadian wild = light green, Scottish domestic = dark blue, and Scottish wild = light blue. (b) Admixture analysis of the Atlantic salmon populations. The analysis was performed using the program ADMIXTURE with *K* = 4, based on 55,406 polymorphic markers. Each color represents a different theoretical ancestral population and each vertical line represent a single individual

The PCA using 55,406 polymorphic SNPs revealed four distinct clusters corresponding to the wild Canadian (Can‐W), wild Scottish (Sct‐W), domesticated Canadian (Can‐D), and domesticated Scottish populations (Sct‐D; Figure 1a). Principal components (PCs) 1 and 2 jointly accounted for 29.6% of total variance, with PC 1 (23.8%) separating Canadian and Scottish populations. Then, PC 2 (5.8%) discriminated Sct‐W, Sct‐D, and Can‐W populations as well as some individual clusters existing within the Can‐W population. Regarding this axis, Can‐D individuals formed a more heterogeneous group as some individuals are geometrically spread along PC 2, with even three individuals being distant from the main cluster. In agreement with PCA results, ADMIXTURE revealed the same four clusters (Figure 1b), based on the lowest cross‐validation error. As ADMIXTURE analysis does not account for the bias generated by MAF filtering, we conducted this analysis using all SNPs, finding four distinctive clusters corresponding to the four original populations, which is congruent with the results including MAF filtering.

### 3.3 Genomic markers putatively under selection distributed across the genome

#### 3.3.1 Bayesian *F*_ST_ method

**Figure 2:**
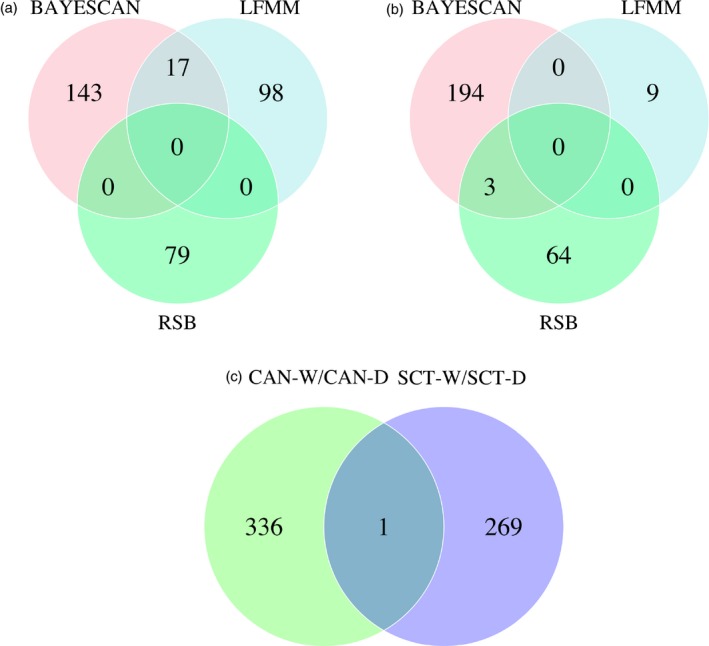
Venn diagrams showing shared SNPs with evidence of selection among three independent tests in Canada populations (a) and Scottish populations (b). Also shown is the overlap of identified loci between both Canada and Scotland across all three tests (BayeScan, LFMM, Rsb [c])

**Figure 3:**
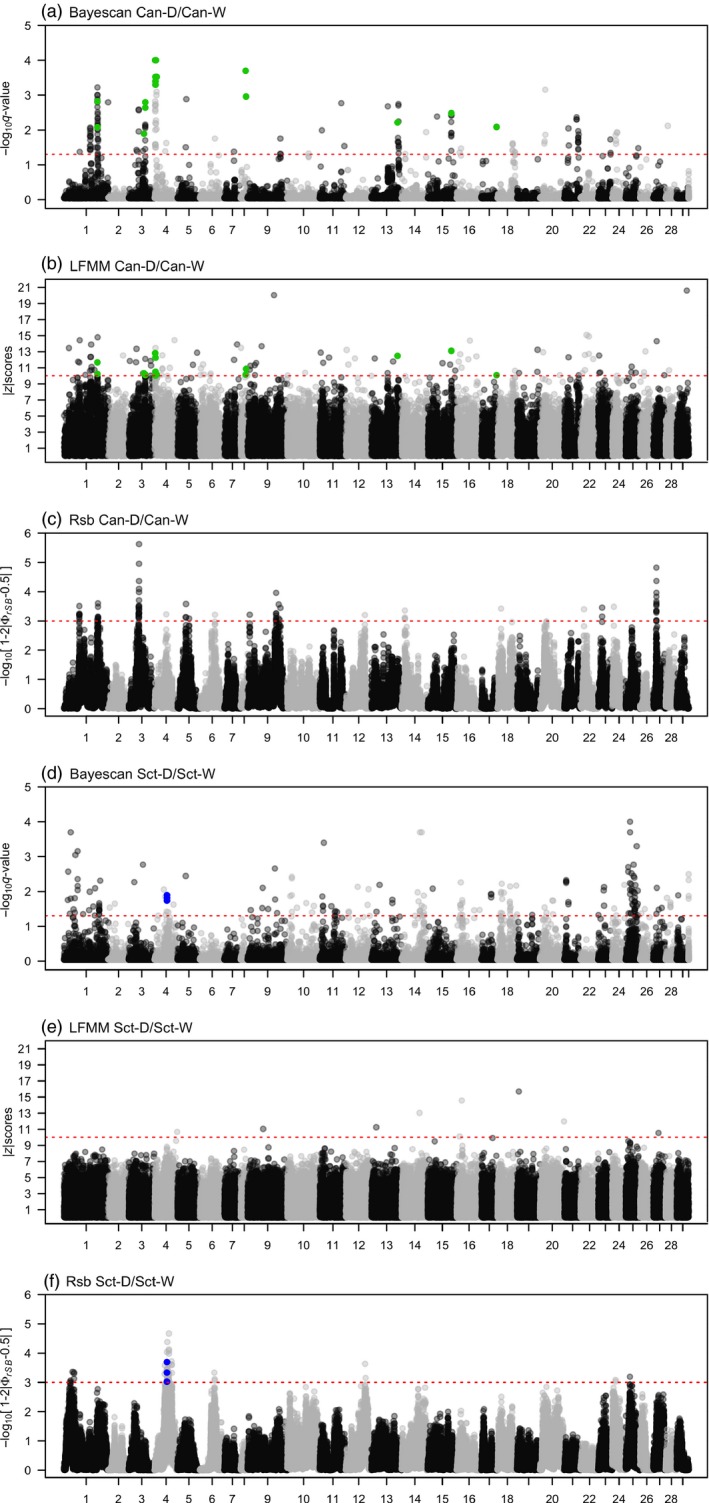
Manhattan plots showing the results of the BayeScan, LFMM, and Rsb outlier analyses applied across the domesticated strain/wild population pairs for Canada (Can; a–c) and Scotland (Sct; d–f). Salmon linkage groups were mapped are indicated on the *x*‐axis. The dashed red line indicates the significant threshold applied to detect the outlier SNPs from the entire dataset. In BAYESCAN analysis this line represents the *q*‐value threshold at log_10_(0.05) = 1.32, which corresponds to Bayes factor of 10. In LFMM, the threshold was set at *z*‐score = 10, while *Rsb* significant threshold was set at −log_10_ one‐tailed *p*‐value = 3. Green dots in Canadian populations are the SNP common between BAYESCAN and LFMM analysis. In the case of Scottish population, blue dots are loci shared between BAYESCAN and Rsb

In Canadian populations, Bayescan analysis detected 160 SNPs putatively under divergent selection (Figure 2a). These markers were distributed among almost all the chromosomes (Figure 3a). The largest chromosome, Ssa01, showed 42 loci above the significance threshold, of which 22 markers had −log_10_ ≥ 2, meaning that these markers are potentially decisive evidence of selection. This chromosome presented the highest number of outliers concentrated within two regions, one of 370 Kb (13 SNPs) and the second one of 2,672 Kb (26 SNPs), respectively. Chromosomes Ssa03 and Ssa04 also contained, respectively, 15 and 27 loci potentially under divergent selection, the majority with a −log_10_ ≥ 2. Other chromosomes with several outlier markers were Ssa13, Ssa15, and Ssa21, with 13, 10, and 15 loci, respectively.

In Scottish populations, Bayescan detected 197 outliers SNP distributed along the vast majority of the chromosomes, similarly to what we observed for Canadian populations (Figure 2b). Yet, in contrast to the analysis in Canadian populations, outliers were distributed in larger regions and were more evenly spaced within a given chromosome (Figure 3d). For instance, chromosome Ssa01 presented 30 outliers located in spanning regions of only 11 to 6,689 Kb long. The chromosome Ssa25 showed the highest number of outliers with 38 in total. Up to 20 outliers were also found in Ssa01, Ssa14, and Ssa18 chromosomes. A summary of Bayescan results for both populations is shown in Table 2.

**Table 2:** Results of Bayescan analysis showing SNPs identified by chromosome

Chromosome	Can‐D/Can‐W					Sct‐D/Sct‐W				
	Initial SNPs number	Strong	Very strong	Decisive	Total	Initial SNPs number	Strong	Very strong	Decisive	Total
Ssa01	4,649	7	13	22	42	10,510	13	8	9	30
Ssa02	1,687					3,341		2		2
Ssa03	1,821	4	3	8	15	5,571			2	2
Ssa04	1,839	2	5	20	27	3,954	4	5	1	10
Ssa05	2,658		1	1	2	5,237			1	1
Ssa06	1,759		1		1	4,672				
Ssa07	1,448	1			1	3,836				
Ssa08	525			2	2	1,243				
Ssa09	4,166	2	1		3	7,981	3	2	2	7
Ssa10	3,626	1			1	6,882	2	5	2	9
Ssa11	2,590		2	1	3	5,400	4	4	1	9
Ssa12	3,027					5,440	2	1	2	5
Ssa13	2,902	2	6	5	13	5,811	1	2	1	4
Ssa14	2,683	1	1		2	5,583	8	5	2	15
Ssa15	2,674	1	4	5	10	6,150			1	1
Ssa16	2,547	2			2	4,669	4	5	1	10
Ssa17	1,501			1	1	2,696		3		3
Ssa18	1,954	4	3		7	4,037	5	8	3	16
Ssa19	2,269					4,625	1			1
Ssa20	2,581		3	1	4	5,022	2	3		5
Ssa21	1,172	2	9	4	15	2,894		2	4	6
Ssa22	2,076					3,921				
Ssa23	1,369	1	1		2	2,995	1	1	2	4
Ssa24	1,384		5		5	2,982	1	1	1	3
Ssa25	1,569	1			1	3,316	9	13	16	38
Ssa26	765					2,003	2			2
Ssa27	1,014					2,873	2	1	1	4
Ssa28	744			1	1	2,042		5		5
Ssa29	1,307					2,982		1		1
Unknown	893					1,918				4
Total	61,199	31	58	71	160	130,586	64	79	54	197

#### 3.3.2 LFMM method

We considered a |*z*| scores higher than 10 as the threshold to select SNPs associated with domestication using this approach. This cutoff value indicated significant SNP effect at the level of *p*‐value < 10e‐7 after applying a standard Bonferroni correction for *α* = 0.05. We found 115 outliers in Canadian populations (Figure 2a) and nine outliers in Scottish populations (Figure 2b). In Canadian populations, these outliers were distributed in all chromosomes, except for Ssa14 chromosome (Figure 3b). The most representative chromosomes were Ssa01, Ssa03, and Ssa04 with 15, 11, and 14 outlier markers, respectively. In Scottish populations, the nine outliers found were distributed in eight chromosomes with Ssa16 being the only chromosomes harboring two outliers (Figure 3e). Details of these results are shown in Table 3.

**Table 3:** Results of LFMM and Rsb Analyses SNPs identified by chromosome

Chromosome	Can‐D/Can‐W			Sct‐D/Sct‐W		
	Initial SNPs number	LFMM	Rsb	Initial SNPs number	LFMM	Rsb
Ssa01	4,649	15	16	10,510		6
Ssa02	1,687	1		3,341		
Ssa03	1,821	11	21	5,571		
Ssa04	1,839	14	1	3,954	1	53
Ssa05	2,658	2	4	5,237		
Ssa06	1,759	6	1	4,672		3
Ssa07	1,448	2		3,836		
Ssa08	525	4		1,243		
Ssa09	4,166	7	12	7,981	1	
Ssa10	3,626	2		6,882		
Ssa11	2,590	3		5,400		
Ssa12	3,027	3	1	5,440		2
Ssa13	2,902	4		5,811	1	
Ssa14	2,683		4	5,583	1	
Ssa15	2,674	4		6,150		
Ssa16	2,547	6		4,669	2	
Ssa17	1,501	1		2,696		
Ssa18	1,954	1	1	4,037		
Ssa19	2,269	3		4,625	1	
Ssa20	2,581	2		5,022	1	
Ssa21	1,172	2		2,894		
Ssa22	2,076	7	1	3,921		
Ssa23	1,369	3	2	2,995		
Ssa24	1,384	1	1	2,982		2
Ssa25	1,569	3		3,316		1
Ssa26	765	2		2,003		
Ssa27	1,014	2	14	2,873	1	
Ssa28	744	1		2,042		
Ssa29	1,307	1		2,982		
Unknown	893	2		1,918		
Total	61,199	115	79	130,586	9	67

#### 3.3.3 Rsb method

The Rsb analysis between Canadian wild and domestic populations revealed 79 SNPs under selection (Figure 2a), distributed on 13 chromosomes (Figure 3c). The highest scores were found in Ssa03 where 21 SNPs passed the threshold of −log_10_(*p*‐value) equal to three within a region of 1,269 Kb long. The region with the second highest score was on Ssa27 with 14 significant markers inside a 280 Kb region. For the comparison between wild and domestic in Scottish populations, Rsb detected 67 SNPs under selection (Figure 3f). The most representative chromosome was Ssa04 with 53 markers located within a region of 26,190 Kb long. Other significant SNPs are detailed in Table 3.

### 3.4 Genomic regions putatively under selection distributed across the chromosomes

**Figure 4:**
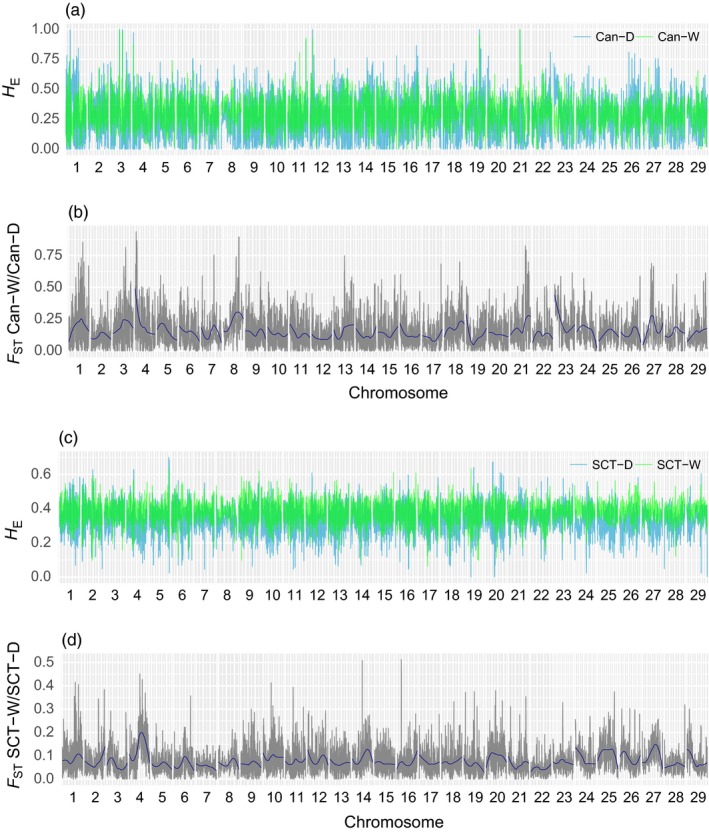
Kernel‐smoothing *F*_ST_ calculated in 500‐kb with steps of 50 kb. (a) Pattern of heterozygosity in domestic (blue) and wild (green) Canadian populations. (b) Pattern of *F*_ST_ between domestic/wild Canadian populations. (c) Pattern of heterozygosity in domestic (blue) and wild (green) Scottish populations. (d) Pattern of *F*_ST_ between domestic/wild Scottish populations

**Figure 5:**
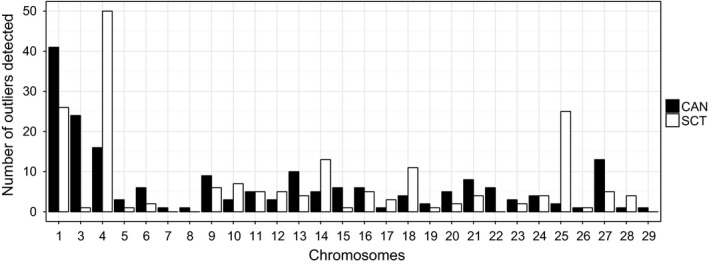
Distribution of the number total of outliers along the Atlantic salmon chromosome considering Canadian (CAN in black) and Scottish population (SCT)

Overall, kernel‐smoothing *F*_ST_ results did not reveal any obvious island of divergence in common between wild versus farmed salmon in both part of the Atlantic (i.e., Canada and Scotland; Figure 4). Accordingly, outliers found in each population were distributed differently along the chromosomes (Figure 5). For instance, more than 24 outliers were found as belonging to Ssa03 chromosome in Canadian populations, whereas only one was located in Ssa03 for the Scottish population. Conversely, 25 outliers were detected on Ssa25 in the Scottish populations and only two for the Canadian ones. Ssa01 and Ssa04 were the only chromosomes showing a high number of outliers detected in both systems (Figure 5). Yet, the chromosomes that harbored the highest number of outliers were different for the Canadian populations and Scottish populations; Ssa01 and Ssa04 respectively.

### 3.5 Genomic footprints of selection: Inconsistencies among methods

We uncovered a total of 337 and 270 SNPs putatively under divergent selection for Canadian and Scottish populations, respectively, according to the three complementary methods (Figure 2c). Overlaps are summarized in Venn diagrams in Figure 2c. In Canadian populations, only Bayescan and LFMM approaches jointly identified 17 outlier SNPs (Figures 2a and 3), whereas for Scottish population, only Rsb and Bayescan approaches found three outlier SNPs in common (Figures 2b and 3). Among these 17 shared outliers between Bayescan and LFMM in Canadian populations, seven regions were found to belong to seven genes (*gdnf; opn4x1b2; svil‐like; tbck‐like; plxnb2‐like* and two uncharacterized proteins) in Atlantic salmon genome annotation. In Scottish populations, three shared SNPs belong to two predicted genes (*brwd3‐like* and *sh3bgrl3‐like*). Only one common outlier on chromosome 25 was shared between the two geographical origins (Figure 2c), even when we considered all the 337 and 270 outliers detected. This common outlier (Affx‐87919237) is located in the gene coding for ubiquitin‐conjugating enzyme E2F putative (*ube2f*).

### 3.6 Investigating for parallel signal of selection among the North American and European lineages

Overall, we found a total of 138 and 121 genes among the 337 and 270 outlier sequences detected as being potential targets of selection between wild versus domesticated Atlantic salmon, for Canada and Scotland, respectively. Among these sets of genes, we discovered four genes overlapping continents: ubiquitin‐conjugating enzyme E2F putative (*ube2f*), collagen alpha‐1XIII chain (*coda1*), autism susceptibility 2 protein‐like (LOC106585083), and transient receptor potential cation channel subfamily M member 3‐like (LOC106580056). A list of the most important genes is described in Tables 4 and 5 for Canadian and Scottish populations, respectively, while the complete list of genes is available in Supporting Information Tables S1 and S2. A gene ontology (GO) enrichment analysis on the total of outlier sequences found in both populations revealed that 11 and 12 GO categories were significantly over‐represented in Canadian and Scottish populations, respectively. The most representative categories in both populations were cellular and metabolic processes (GO:0009987 and GO:0008152) including 38.8%–51.4% and 33.9%–34.7% of the genes, respectively. Other categories were biological regulation, developmental processes, localization, multicellular organismal processes, response to stimulus, biological adhesion, immune system processes, and reproduction. All these categories were shared in both populations, although locomotion category was only present in Scottish population (this includes 0.9% of the genes).

**Table 4:** Summary of genomic regions under selection and associated genes in Canadian populations

Chromosome	Position SNPs	Test	Gene	Protein name
Ssa01	118333490	RSB	LOC106563782	PREDICTED: unconventional myosin‐Vb‐like
Ssa01	119321920	LFMM_BYS	opn4x1b2	Melanopsin
Ssa01	119472753	LFMM_BYS	gdnf	Glial cell‐derived neurotrophic factor
Ssa01	120337869 120350430	BYS	LOC106564357	PREDICTED: polypeptide N‐acetylgalactosaminyltransferase 9‐like
Ssa03	35341493 35353272	RSB	LOC106600280	PREDICTED: matrix metalloproteinase‐14‐like
Ssa03	58772401	LFMM_BYS	LOC106600794	PREDICTED: supervillin‐like
Ssa03	58814947	LFMM_BYS	LOC106600792	PREDICTED: uncharacterized protein LOC106600792
Ssa04	1988842	LFMM_BYS	LOC106602233	PREDICTED: uncharacterized protein LOC106602233
Ssa04	2491374 2503265	BYS	LOC106602228	PREDICTED: dual specificity testis‐specific protein kinase 2‐like
Ssa04	41701375	RSB	LOC106603279	PREDICTED: unconventional myosin‐XVIIIa‐like
Ssa04	54112595	LFMM	LOC106603471	PREDICTED: collagen alpha‐1(XXVI) chain‐like
Ssa06	37003521	LFMM	LOC106607315	PREDICTED: unconventional myosin‐X‐like
Ssa08	19722947	LFMM_BYS	LOC106610360	PREDICTED: TBC domain‐containing protein kinase‐like protein
Ssa17	54473681	LFMM_BYS	LOC106576598	PREDICTED: plexin‐B2‐like
Ssa18	13286009	RSB	coda1	Collagen alpha‐1XIII chain
Ssa18	62851227	BYS	LOC106577990	PREDICTED: E3 ubiquitin‐protein ligase TRIM39‐like
Ssa20	15597033	BYS	LOC106580056	PREDICTED: transient receptor potential cation channel subfamily M member 3‐like
Ssa22	29365659 29365735	LFMM	LOC106583145	PREDICTED: collagen alpha‐1(VII) chain‐like
Ssa24	12213523	BYS	LOC106585083	PREDICTED: autism susceptibility gene 2 protein‐like
Ssa25	19817836	LFMM	ube2f	Ubiquitin‐conjugating enzyme E2F (putative)
Ssa27	10232307 10238834	RSB	brd2	Bromodomain containing 2
Ssa27	10251922	RSB	LOC106588396	PREDICTED: collagen alpha‐2(XI) chain‐like

**Table 5:** Summary of genomic regions under selection and associated genes in Scottish populations

Chromosome	Position SNPs	Test	Gene	Protein name
Ssa01	48495280 48506349	BYS	LOC106604517	PREDICTED: myopalladin‐like
Ssa03	50571649	BYS	LOC106600700	PREDICTED: transient receptor potential cation channel subfamily M member 4‐like
Ssa04	44415367	BYS_RSB	LOC106603326	PREDICTED: SH3 domain‐binding glutamic acid‐rich‐like protein
Ssa04	44432033 44436726	BYS_RSB	LOC106603328	PREDICTED: bromodomain and WD repeat‐containing protein 3‐like
Ssa04	45658450 45667448	RSB	LOC106603377	PREDICTED: long‐chain fatty acid–CoA ligase 4‐like
Ssa04	47256953 47265274	RSB	LOC106603586	PREDICTED: E3 ubiquitin‐protein ligase Siah2
Ssa04	51300396 51426545	RSB	LOC106603511	PREDICTED: active breakpoint cluster region‐related protein‐like
Ssa10	16255371 16284803	BYS	LOC106613666	PREDICTED: unconventional myosin‐IXb‐like
Ssa14	47278390	BYS	tekt2	Tektin 2 (testicular)
Ssa14	65526017	BYS	LOC106570217	PREDICTED: collagen alpha‐1(IX) chain‐like
Ssa14	65541964 65552172	BYS	LOC106570216	PREDICTED: collagen alpha‐1(XXII) chain‐like
Ssa14	79347457 79378912	BYS	LOC106570512	PREDICTED: collagen alpha‐2(IX) chain‐like
Ssa18	13282526 13283308	BYS	coda1	Collagen alpha‐1XIII chain
Ssa18	47314912	BYS	LOC106577539	PREDICTED: neurexin‐2‐like
Ssa20	15586930	BYS	LOC106580056	PREDICTED: transient receptor potential cation channel subfamily M member 3‐like
Ssa24	12125145 12127325	RSB	LOC106585083	PREDICTED: autism susceptibility gene 2 protein‐like
Ssa25	19817836	BYS	ube2f	Ubiquitin‐conjugating enzyme E2F (putative)
Ssa25	32313916 32325227	BYS	mspd2	Motile sperm domain‐containing protein 2
Ssa27	18583143	BYS	LOC106588644	PREDICTED: zona pellucida sperm‐binding protein 3‐like

## 4 DISCUSSION

Understanding how the process of domestication may shape the genome of wild to domesticated animals is particularly useful for enhancing our knowledge on how human‐driven selection may induce genetic changes as it may provide a practical framework to guide genetic improvement practices (Wang, Xie, Peng, Irwin, & Zhang, 2014). Here, we performed a large genomewide scan, using a dense SNP array and three outlier methods, to investigate genomic footprints of domestication in two geographically isolated Atlantic salmon strains. Overall, our results revealed only a few footprints of domestication that overlap among methods and populations, suggesting that detection of selection depends on the method used and parallel genomic changes resulting from domestication events are rare in this system. Indeed, domestication is a recent and ongoing process in Atlantic salmon populations, which may explain this low number of genomic changes. Furthermore, as Canadian and Scottish populations have diverged independently for possibly as much as 1 million years (Rougemont & Bernatchez, 2018), it is likely that this had led to different standing genetic variation upon which selection may be acting, limiting the potential for parallel evolution at the genome level in this study system. This is in agreement with previous population genomic work on Atlantic salmon (Mäkinen et al., 2014; Vasemägi et al., 2012) and, more broadly, on several studies showing that selection may act faster on standing variation compared to new mutations (Barrett & Schluter, 2008). Limited evidence for parallel impact of artificial selection at the genome level contrasts with the pronounced pattern of parallelism previously documented at the transcriptome level (Roberge et al., 2006), suggesting the different genomic architecture may result in similar pattern of gene expression as well as parallel phenotypic changes occurring during domestication. Clearly, shedding light on the complete genomic basis of domestication of Atlantic salmon would require using alternative methods rather than genome scan and collecting a larger dataset with known phenotypes. Future studies testing for parallel evolution in Atlantic salmon should consider this avenue.

### 4.1 Limitations of genome scan methods

Traits of interest selected in aquaculture programs, such as growth and fat content, may be polygenic, that is, controlled by many genes with high variance of effect size (Gagnaire & Gaggiotti, 2016). Polygenic selection may imply a subtle change of allele frequencies at several loci that would be hard to detect using genome scan methods that mainly focus on moderate to high shift in allele frequency at few independent loci. For instance, Bourret, Dionne, and Bernatchez (2014) genotyped 5,568 SNPs to test for differential allelic and genotypic frequencies between juveniles (smolts) migrating to sea and adults (grilses) returning to freshwater after 1 year at sea. Although numerous outliers were identified by the single‐locus analysis, no evidence for parallel, temporally repeated selection was found. In contrast, the multilocus approach detected repeated patterns of selection for a multilocus group of 34 co‐varying SNPs. Overall, the large difference in the sampling design of wild populations between Canada and Scotland may partly explain why so few common outliers were highlighted by genome scan methods when comparing the two locations. The minor allele frequency and Hardy–Weinberg filtering could also have affected the final result of the analysis, leading to lower number of SNPs detected. However, Bayescan analysis conducted with no MAF and HWE filters still revealed none loci putatively selected in Canadian populations, although 104 markers (Supporting Information Table S3) were detected in Scottish population, which is a lower number than those detected using filtering options, revealing the suitableness of applying these filters to discard low‐quality genotypes.

Alternative models to common genome scan approaches, such as a quantitative genetic framework, provide powerful tools to investigate polygenic selection. Earlier Atlantic salmon studies of quantitative trait loci (QTL) mapping have already reported a large number of different QTLs for growth (Baranski, Moen, & Våge, 2010; Gutierrez et al., 2012; Houston et al., 2009; Reid, Szanto, Glebe, Danzmann, & Ferguson, 2005), although genomewide studies revealed very low levels of association between markers and growth (Gutierrez, Yáñez, & Davidson, 2015; Gutierrez, Yáñez, Fukui, Swift, & Davidson, 2015). This indicates that growth‐related traits are likely to be controlled by a number of population‐specific loci of low to moderate effect with an important polygenic component (Tsai et al., 2015), making it difficult to compare our results with those obtained from GWAS or QTL mapping. Future studies combining population genomic and quantitative genetic framework would then provide a more inclusive approach for uncovering the molecular footprints of domestication in Atlantic salmon. Furthermore, the complete functional annotation of the Atlantic salmon genome will undoubtedly enhance opportunities for a comprehensive understanding of the effects of domestication in Atlantic salmon populations (Macqueen et al., 2017).

### 4.2 Standing genetic variation and loss of genetic diversity

Of the 151,509 SNPs successfully genotyped, 61,199 and 130,586 SNPs were polymorphic for Canadian and Scottish populations, respectively. This striking difference on the number of polymorphic SNPs is in accordance with earlier studies based on various types of markers (mtDNA, microsatellites, AFLPs, SNPs) showing that North American Atlantic salmon populations have lower genetic diversity than European populations (Bourret et al., 2013; Mäkinen et al., 2014). This may also be explained to some extent by ascertainment bias as the 200K SNP Array used in the present study was created based on genomic information mostly from European salmon (Yáñez et al., 2016). Both lineages only shared 55,406 SNPs in common and the genetic diversity analysis of these SNPs revealed that the lowest and highest levels of heterozygosity were found in Canadian domestic and Scottish wild population, respectively. Indeed, lower levels of genetic diversity in both domestic populations compared to their wild homologs were expected as most of domestic populations experience an inevitable loss of genetic diversity due to both selective breeding as well as the absence of genetic connectivity with other populations as found in nature (Baumung, Simianer, & Hoffmann, 2004; Johansson & Rendel, 1968). Concomitantly, and as expected from reduced diversity within population, domesticated populations showed a higher proportion of genetic differentiation (*F*_ST_ = 0.393) than wild populations (*F*_ST_ = 0.286). This outcome is in agreement with the work of Mäkinen et al. (2014) who reported that the mean value of genetic differentiation between two populations of hatcheries strain is higher than between wild populations. On the other hand, the low level of genetic differentiation (*F*_ST_ = 0.171) between domestic and wild Scottish populations showed that using a wild population from a nearby geographical region as a genetic proxy was appropriate, in the absence of a true ancestral population.

### 4.3 Detecting the impacts of domestication with genome scans

Using a genomewide SNP array, we found limited evidence of potential signatures of selection that were jointly detected between domestic and natural populations, in Canada and Scotland, considering the outcomes of three different outlier tests. This lack of congruence among methods is likely to result from the statistics underlying each approach: LFMM is based on linear associations, Bayescan is an *F*_ST_ differentiation method, and Rsb is a haplotypes comparison method. As LFMM is based on linear model and it detects a few numbers of outliers in our study system, this approach may not be the most appropriate approach for this kind of experimental design. More particularly, Rsb test is likely to detect more recent selection signatures in comparison with *F*_ST_‐based methods (Oleksyk, Smith, & O'Brien, 2009). On the other hand, Bayescan was also the method that resulted in the highest number of outliers identified but it should be noted that we used a permissive threshold (PO = 10), as used by Gutierrez, Yáñez, and Davidson (2015); Chen et al. (2016); Lin et al. (2017) that may have led to a high rate of false positives (Lotterhos & Whitlock, 2015). This nonoverlap in outlier results tests has already been observed and discussed by Mäkinen et al. (2014) who also investigated genomic signal of domestication in Atlantic salmon using Bayescan and LnRH methods. As the three different tests can detect different patterns of molecular variation (de Simoni Gouveia, da Silva, Paiva, & de Oliveira, 2014) and at different time scales (Hohenlohe et al., 2010; Oleksyk et al., 2009) even if SNPs are only detected by one test, they still can be real targets of selection. Otherwise, it is important to mention that chromosomal differences between European and North American Atlantic salmon lineages can have an impact on analyses based on haplotype measures (Rsb). According to the linkage map of 3K SNPs, gross chromosomal re‐arrangements between European and North American Atlantic salmon have been identified involving Ssa01 (that presents a translocation of its p arm to Ssa23) and two chromosomal fusions: Ssa26 with Ssa28 and Ssa08 and Ssa29 (Brenna‐Hansen et al., 2012). Nevertheless, while these major structural differences were important, marker order within these chromosomal regions was remarkably conserved (Brenna‐Hansen et al., 2012). Chromosomal re‐arrangements may lead to spurious results when using European Atlantic salmon as the reference genome sequence in North American populations. In this study, the only chromosome presenting re‐arrangements in Canadian populations that harbors loci putatively under selection was Ssa01 with 16 markers; consequently, these results should be interpreted with caution.

### 4.4 Investigating the existence of parallel genomic footprints of Atlantic salmon domestication

Over the past 50 years, intense artificial selection in Atlantic salmon populations has led to the development of strains specialized in certain phenotypic traits which has caused large phenotypic and genotypic changes between wild and domestic populations (Glover et al., 2009). Despite Atlantic salmon populations having been intensely selected for growth‐related traits on both sides of the Atlantic ocean, we found little overlap between the outlier genomic regions identified in previous studies of other Atlantic salmon strains selected for high growth rate (Gutierrez, Yáñez, & Davidson, 2015; Gutierrez, Yáñez, Fukui, et al., 2015). These population‐specific signals suggest that selection may have acted upon different genes, which was already shown by one study documenting genomewide footprints of pig domestication (Amaral et al., 2011). More particularly, several population genomic studies of Atlantic salmon populations have suggested that the same phenotype may arise from different genetic pathways among geographically isolated populations (Elmer et al., 2014; Mäkinen et al., 2014; Perrier, Bourret, Kent, & Bernatchez, 2013; Pujolar, Ferchaud, Bekkevold, & Hansen, 2017; Vasemägi et al., 2012). Evolution of complex parallel phenotypes can indeed arise from different evolutionary routes and this is likely to happen when inbreeding and genetic drift play a greater role than selection.

### 4.5 Parallel genomic regions detected as being putatively under artificial selection

Despite limited evidence for parallelism at the genome level, evidence for parallel evolution was observed at a few potentially important genes. Here, genomic regions harboring collagen alpha‐1XIII chain gene (*coda1*) were identified in both comparisons. There is evidence that collagen genes may be involved in the response of Atlantic salmon to sea lice as initiators of inflammatory cytokine signaling (Castillo‐Briceño et al., 2009; Correa et al., 2016; Krasnov, Skugor, Todorcevic, Glover, & Nilsen, 2012). Therefore, this gene might be involved in the immune response to cope with specific diseases present in aquaculture environment and may be a relevant target of selection. Despite none of the farmed populations having been directly selected for traits associated with disease resistance, farmed populations are often subjected to high levels of pathogens, such as parasite infections, which are known to be among the strongest selective forces driving the evolution of host populations (Roberge et al., 2006; Zueva et al., 2014). A second common gene was ubiquitin‐conjugating enzyme E2F putative (*ube2f*), coding for ubiquitin‐conjugating enzyme E2‐F. Growth and development of skeletal muscle undergo breakdown and replacement of proteins during different periods, and in teleost fish, this process involves E2‐ubiquitin‐conjugating enzymes and E3‐ubiquitin ligases (Johnston, Bower, & Macqueen, 2011). Therefore, this gene could be involved in muscle development and growth in Atlantic salmon, which is an important trait selected for enhancing salmon aquaculture production. This gene family was also suggested to be potentially involved in immune response of marine vertebrates (Núñez‐Acuña, Aguilar‐Espinoza, Chávez‐Mardones, & Gallardo‐Escárate, 2012), and more particularly, ubiquitin genes have already been detected to be potential targets of selection in the domestication process of shrimp (Rotllant et al., 2015), auroch (Braud et al., 2017) and rice (Li & Li, 2014). Finally, the autism susceptibility 2 protein‐like (LOC106585083) was also found to overlap between continents. This gene (*Auts2‐like*) has been associated with autism and mental retardation in humans (Bedogni et al., 2010; Oksenberg & Ahituv, 2013). Interestingly, *Auts2* has been found to be under selection in domestic cattle breeds (Consortium, Bovine HapMap, 2009), suggesting that domestication may act on behavioral traits in salmon as in other domestic animals (Clutton‐Brock, 1999). Finally, a potential parallel signal of selection was located in the transient receptor potential cation channel subfamily M member 3‐like (LOC106580056), which belongs to *Trpm3*, a gene associated with the reception of noxious temperature stimuli in mammals (Vriens et al., 2011). Transient receptor potential cation channel subfamily genes are involved in melanocyte pigmentation (Cieslak, Reissmann, Hofreiter, & Ludwig, 2011). Therefore, this gene may play a role in the immune system of domesticated Atlantic salmon and Kittilsen, Johansen, Braastad, and Øverli (2012) showed that pigmentation seems to be correlated with the development of the ectoparasitic lice in Atlantic salmon. Interestingly, Yang, Li, Li, Fan, and Tang (2014) also detected selection signature linked to domestication process in this gene by comparing Chinese indigenous and commercial pig breeds.

### 4.6 Biological function of nonparallel genomic regions identified as putatively being under artificial selection

Some of the genes that overlap between statistical methods in Canadian populations (Bayescan and LFMM) included supervillin‐like gene, which is a protein involved in actin and myosin II assembly that promotes cell growth (Fang & Luna, 2013). This gene has been shown to be involved in muscle fiber type determination in large white pigs (Zhu et al., 2016). We suggest that this gene may be associated with muscle development in Atlantic salmon and be under selection for improving growth‐related traits. We also identified *plexin‐b2‐like*, which in mammals is involved in several processes during development of the nervous, cardiovascular, renal, and skeletal system (Worzfeld et al., 2014). Another plexin from the same subfamily has been associated with behavior‐related traits, such as tameness and temperament in rat and cattle (Friedrich, Brand, & Schwerin, 2015; Heyne et al., 2014). This finding is in line with the identification of the gene autism susceptibility 2 protein‐like and both suggest that domestication could be acting on behavior‐related traits. On the other hand, *opn4x1b2* detected in Canadian populations is a melanopsin that can be involved in signaling of photoperiodic information in Atlantic salmon (Sandbakken, Ebbesson, Stefansson, & Helvik, 2012), indicating some role in the adaptation to the aquaculture environment.

We detected a potential gene candidate in Scottish populations, *brwd3*, that has been associated to mental retardation in humans (Field et al., 2007) and which was also identified as a target of selection during the domestication process in cattle (Consortium, Bovine HapMap, 2009). Other genes detected in this study included *myopalladin‐like* gene in Scottish populations, which has been associated with late maturation in Atlantic salmon (Gutierrez et al., 2014). The long‐chain fatty acid–CoA ligase 4‐like gene, located in Ssa04, which belongs to *Acsl* family of genes, was found as a potential gene influenced by domestication in Scottish populations. This gene is involved in lipid metabolism, although the activation of long‐chain fatty acids for synthesis and degradations of cellular lipids (Golej et al., 2011). In rainbow trout, lipid metabolism has been reported to be associated with growth (Xu et al., 2011). Similarly, another gene from the same family, the long‐chain fatty acid‐CoA ligase 1 has been shown to be associated with growth in clam (*Meretrix meretrix*, Dai, Huan, Wang, Xia, & Liu, 2015).

No other overlaps were found with previous studies related to sexual maturity in Atlantic salmon, for both Canadian and Scottish populations. Nevertheless, we found putative outliers in the bromodomain‐containing two genes (*br2*) and the zona pellucida (ZP) sperm‐binding protein 3‐like gene (*Zp3*) in Canadian and Scottish populations, respectively, both localized in chromosome Ssa27. The bromodomain‐containing 2 gene may be involved in spermatogenesis or folliculogenesis (Rhee, Brunori, Besset, Trousdale, & Wolgemuth, 1998), while ZPs are responsible for the initial sperm binding and the subsequent induction of the acrosome reaction that allows sperm penetration in mammals (Lin, Roy, Yan, Burns, & Matzuk, 2007). Thus, these genes are interesting functional candidates for reproduction‐related traits under selection on these populations. To better understand the molecular functions of these genes, we investigated their GO classification. Many genes were categorized into cellular process and metabolic process. Both categories can involve cell growth or anabolic/catabolic process resulting in cell growth, which suggest that these genes could be associated with growth in Atlantic salmon. Admittedly, these observations need to be explored and verified in further studies.

## 5 CONCLUSION

Domestication events in Atlantic salmon have produced pronounced phenotypic changes in domesticated fish compared to their wild counterpart, and the selection process has left important signals throughout the genome of these populations. This study highlighted the existence of nonparallel and parallel signatures of selection between domesticated versus farmed fish, detected by different statistical approaches. A small number of parallel genomic regions putatively under selection belong to genes with molecular functions that might be associated with traits under domestication, such as behavior, immune response, and reproduction. However, the vast majority of genomic regions detected as being putatively under selection are not shared either among method or lineages, suggesting that domestication may involve a different set of genes, perhaps because of the distinct evolutionary history and very different standing genetic variation of Canadian and Scottish populations. Another possibility is that alternative methods to genome scan that test for genomic signal of polygenic adaptation are required for revealing genetic architecture underlying trait of interest. Indeed, the most important trait for which Atlantic salmon has been artificially selected is growth, whereas few selection signatures possibly associated with this trait have been detected, perhaps due to the polygenic nature of this trait. Nevertheless, our results contribute to the detection of variants that may underlie important traits of practical interest for aquaculture and to fully understanding the early patterns and effect of domestication in Atlantic salmon.

## CONFLICT OF INTEREST

None declared.

## DATA ARCHIVING

Data available from the Dryad Digital Repository: https://doi.org/10.5061/dryad.60b9p56.
