# Supplementary material for: Comparing genomic signatures of domestication in two Atlantic salmon (Salmo salar L.) populations with different geographical origins
Source: Evol Appl. 2018 Dec 7;12(1):137–56. doi: 10.1111/eva.12689 (PMC6304691; doi:10.1111/eva.12689)
Supplement: Supplementary file 1 [file EVA-12-137-s001.docx]

Supplementary Material:

| Table S1. Genes identified in Canadian populations, using Atlantic salmon annotation (GCF_000233375.1), which is a complete representation of the genome constructed by using RNA sequencing (RNA-seq) and expressed sequence tags (ESTs) that were mapped to the PFAM, stickleback and zebrafish databases (Lien et al. 2016). | | |
| --- | --- | --- |
| CHROM | **GENE** | **PRODUCT** |
| Ssa01 | LOC106573328 | PREDICTED: protein TSSC1-like |
| Ssa01 | LOC106605956 | PREDICTED: synapse differentiation-inducing gene protein 1-like |
| Ssa01 | enp6 | Ectonucleoside triphosphate diphosphohydrolase 6 |
| Ssa01 | LOC106605990 | PREDICTED: uncharacterized LOC106605990 |
| Ssa01 | LOC106608672 | PREDICTED: cGMP-dependent protein kinase 2-like |
| Ssa01 | LOC106612532 | PREDICTED: CUB and sushi domain-containing protein 1-like |
| Ssa01 | LOC106612512 | PREDICTED: ribosomal protein S6 kinase alpha-2-like |
| Ssa01 | LOC106612469 | PREDICTED: nucleolar RNA helicase 2-like |
| Ssa01 | LOC106611525 | PREDICTED: KIF1-binding protein homolog |
| Ssa01 | LOC106612465 | PREDICTED: serglycin-like |
| Ssa01 | nat9 | N-acetyltransferase 9 (putative) |
| Ssa01 | LOC106612072 | PREDICTED: complement C1q tumor necrosis factor-related protein 1-like |
| Ssa01 | rnf213 | ring finger protein 213 |
| Ssa01 | adk | Adenosine kinase |
| Ssa01 | LOC106611737 | PREDICTED: voltage-dependent anion-selective channel protein 2-like |
| Ssa01 | ctif | CBP80/20-dependent translation initiation factor |
| Ssa01 | LOC106562119 | PREDICTED: solute carrier family 25 member 46 |
| Ssa01 | LOC106563782 | PREDICTED: unconventional myosin-Vb-like |
| Ssa01 | opn4x1b2 | melanopsin |
| Ssa01 | LOC106564089 | PREDICTED: pikachurin-like |
| Ssa01 | gdnf | glial cell derived neurotrophic factor |
| Ssa01 | wdr70 | WD repeat domain 70 |
| Ssa01 | cssa01h5orf42 | chromosome ssa01 open reading frame, human C5orf42 |
| Ssa01 | LOC106564357 | PREDICTED: polypeptide N-acetylgalactosaminyltransferase 9-like |
| Ssa01 | LOC106564312 | PREDICTED: autism susceptibility gene 2 protein-like |
| Ssa01 | LOC106564816 | PREDICTED: solute carrier family 35 member E4-like |
| Ssa01 | LOC106572640 | PREDICTED: low-density lipoprotein receptor-related protein 1B-like |
| Ssa03 | LOC106599664 | PREDICTED: laminin subunit alpha-3-like |
| Ssa03 | LOC106599680 | PREDICTED: zinc finger protein 521-like |
| Ssa03 | cbln3 | cerebellin 3 precursor |
| Ssa03 | LOC106600289 | PREDICTED: sal-like protein 3 |
| Ssa03 | LOC106600280 | PREDICTED: matrix metalloproteinase-14-like |
| Ssa03 | LOC106600278 | PREDICTED: apoptotic chromatin condensation inducer in the nucleus-like |
| Ssa03 | LOC106599941 | PREDICTED: kinetochore protein NDC80 homolog |
| Ssa03 | LOC106599941 | PREDICTED: kinetochore protein NDC80 homolog |
| Ssa03 | LOC106600228 | PREDICTED: GTP-binding protein REM 2-like |
| Ssa03 | LOC106600169 | PREDICTED: ETS-related transcription factor Elf-1-like |
| Ssa03 | LOC106600156 | PREDICTED: endothelin B receptor-like |
| Ssa03 | LOC106600689 | PREDICTED: ATP-sensitive inward rectifier potassium channel 12-like |
| Ssa03 | LOC106600693 | PREDICTED: AP-2 complex subunit alpha-2-like |
| Ssa03 | LOC106600798 | PREDICTED: uncharacterized protein PB18E9.04c-like |
| Ssa03 | LOC106600794 | PREDICTED: supervillin-like |
| Ssa03 | LOC106600793 | PREDICTED: putative all-trans-retinol 13,14-reductase |
| Ssa03 | LOC106600792 | PREDICTED: uncharacterized protein LOC106600792 |
| Ssa03 | LOC106601691 | PREDICTED: small G protein signaling modulator 3-like |
| Ssa04 | LOC106602233 | PREDICTED: uncharacterized protein LOC106602233 |
| Ssa04 | LOC106602231 | PREDICTED: rho GTPase-activating protein 7-like |
| Ssa04 | LOC106602229 | PREDICTED: fatty-acid amide hydrolase 2-A-like |
| Ssa04 | LOC106602228 | PREDICTED: dual specificity testis-specific protein kinase 2-like |
| Ssa04 | LOC106602215 | PREDICTED: GTPase IMAP family member 7-like |
| Ssa04 | LOC106602188 | PREDICTED: zinc finger and SCAN domain-containing protein 12-like |
| Ssa04 | LOC106602720 | PREDICTED: storkhead-box protein 2-like |
| Ssa04 | alg8 | ALG8, alpha-1,3-glucosyltransferase |
| Ssa04 | LOC106602846 | PREDICTED: beta-galactoside alpha-2,6-sialyltransferase 1-like |
| Ssa04 | LOC106603030 | PREDICTED: acidic fibroblast growth factor intracellular-binding protein-like |
| Ssa04 | LOC106603279 | PREDICTED: unconventional myosin-XVIIIa-like |
| Ssa04 | LOC106603282 | PREDICTED: vascular endothelial zinc finger 1-like |
| Ssa04 | LOC106603471 | PREDICTED: collagen alpha-1(XXVI) chain-like |
| Ssa04 | LOC106603846 | PREDICTED: gamma-aminobutyric acid receptor subunit beta-2-like |
| Ssa05 | LOC106604622 | PREDICTED: protocadherin Fat 4-like |
| Ssa05 | LOC106604602 | PREDICTED: uncharacterized LOC106604602 |
| Ssa05 | LOC106605788 | PREDICTED: zinc finger and BTB domain-containing protein 7B-like |
| Ssa06 | LOC106606281 | PREDICTED: uncharacterized LOC106606281 |
| Ssa06 | LOC106606471 | PREDICTED: ras-related protein Rab-26-like |
| Ssa06 | LOC106607315 | PREDICTED: unconventional myosin-X-like |
| Ssa06 | LOC106607293 | PREDICTED: potassium voltage-gated channel subfamily H member 4-like |
| Ssa06 | LOC106607715 | PREDICTED: ankyrin repeat domain-containing protein 6-like |
| Ssa06 | casp8ap2 | caspase 8 associated protein 2 |
| Ssa07 | LOC106609242 | PREDICTED: transcription factor EC-like |
| Ssa08 | LOC106610360 | PREDICTED: TBC domain-containing protein kinase-like protein |
| Ssa09 | LOC106610962 | PREDICTED: transcription factor IIIB 90 kDa subunit-like |
| Ssa09 | LOC106611163 | PREDICTED: stathmin-4-like |
| Ssa09 | LOC106611507 | PREDICTED: protein diaphanous homolog 2-like |
| Ssa09 | LOC106612700 | PREDICTED: phospholipase D3-like |
| Ssa09 | LOC106612696 | PREDICTED: RNA-binding protein Nova-1-like |
| Ssa09 | LOC106612684 | PREDICTED: mitochondrial import inner membrane translocase subunit TIM50-like |
| Ssa09 | bcas3 | breast carcinoma amplified sequence 3 |
| Ssa09 | LOC106612785 | PREDICTED: nucleoredoxin-like |
| Ssa10 | mcoln2 | mucolipin 2 |
| Ssa10 | nox5 | NADPH oxidase 5 |
| Ssa10 | LOC106560901 | PREDICTED: vacuolar protein sorting-associated protein 4A |
| Ssa11 | kif6 | kinesin family member 6 |
| Ssa11 | LOC106561917 | PREDICTED: oxidoreductase HTATIP2-like |
| Ssa11 | cart | Cocaine- and amphetamine-regulated transcript protein |
| Ssa11 | LOC106563422 | PREDICTED: voltage-gated potassium channel subunit beta-2-like |
| Ssa11 | ufm1 | ubiquitin-fold modifier 1 |
| Ssa12 | LOC106564346 | PREDICTED: uncharacterized protein LOC106564346 |
| Ssa12 | LOC106565033 | PREDICTED: recombining binding protein suppressor of hairless-like protein |
| Ssa12 | LOC106565836 | PREDICTED: succinyl-CoA ligase |
| Ssa13 | LOC106567547 | PREDICTED: kin of IRRE-like protein 3 |
| Ssa13 | LOC106567552 | PREDICTED: CAP-Gly domain-containing linker protein 2-like |
| Ssa13 | LOC106568371 | PREDICTED: contactin-associated protein-like 4 |
| Ssa13 | LOC106568376 | PREDICTED: rab GTPase-activating protein 1-like |
| Ssa13 | LOC106568425 | PREDICTED: protein FAM163B-like |
| Ssa13 | LOC106568427 | PREDICTED: uncharacterized LOC106568427 |
| Ssa13 | LOC106568434 | PREDICTED: tyrosine-protein kinase SgK223-like |
| Ssa14 | LOC106568976 | PREDICTED: gelsolin-like |
| Ssa14 | LOC106568975 | PREDICTED: protein wntless homolog |
| Ssa14 | LOC106568937 | PREDICTED: SPRY domain-containing SOCS box protein 4-like |
| Ssa15 | LOC106571386 | PREDICTED: solute carrier family 35 member F1-like |
| Ssa15 | LOC106572340 | PREDICTED: suppressor of cytokine signaling 2-like |
| Ssa15 | LOC106572522 | PREDICTED: inactive dipeptidyl peptidase 10-like |
| Ssa15 | LOC106572535 | uncharacterized LOC106572535 |
| Ssa15 | LOC106572534 | PREDICTED: solute carrier family 26 member 9-like |
| Ssa15 | LOC106572896 | PREDICTED: AMP deaminase 2-like, partial |
| Ssa16 | LOC106573109 | PREDICTED: sodium-dependent neutral amino acid transporter B(0)AT2-like |
| Ssa16 | LOC106573249 | PREDICTED: mitochondrial inner membrane protease subunit 2-like |
| Ssa16 | LOC106573307 | PREDICTED: early endosome antigen 1-like |
| Ssa16 | LOC106573845 | PREDICTED: aspartyl aminopeptidase-like |
| Ssa16 | LOC106574402 | PREDICTED: motile sperm domain-containing protein 2-like |
| Ssa17 | LOC106576598 | PREDICTED: plexin-B2-like |
| Ssa18 | coda1 | Collagen alpha-1XIII chain |
| Ssa18 | LOC106577966 | PREDICTED: nuclear factor 7, brain-like |
| Ssa18 | LOC106577990 | PREDICTED: E3 ubiquitin-protein ligase TRIM39-like |
| Ssa18 | LOC106577967 | PREDICTED: zinc-binding protein A33-like |
| Ssa19 | LOC106578362 | PREDICTED: pro-neuregulin-3, membrane-bound isoform-like |
| Ssa19 | LOC106579654 | PREDICTED: laminin subunit alpha-1-like |
| Ssa20 | LOC106580081 | PREDICTED: proprotein convertase subtilisin/kexin type 5-like |
| Ssa20 | LOC106580056 | PREDICTED: transient receptor potential cation channel subfamily M member 3-like |
| Ssa20 | LOC106580011 | PREDICTED: LIM domain transcription factor LMO4.1-like |
| Ssa20 | LOC106579986 | PREDICTED: nuclear receptor subfamily 6 group A member 1-A-like |
| Ssa20 | LOC106581345 | PREDICTED: protocadherin Fat 3-like, partial |
| Ssa21 | pkp4 | plakophilin 4 |
| Ssa21 | rbms1 | RNA binding motif single stranded interacting protein |
| Ssa21 | LOC106581793 | PREDICTED: beta-1,3-galactosyltransferase 1-like |
| Ssa21 | LOC106582440 | PREDICTED: glypican-6-like |
| Ssa21 | LOC106582447 | PREDICTED: muscleblind-like protein 2a |
| Ssa21 | LOC106582447 | PREDICTED: muscleblind-like protein 2a |
| Ssa21 | LOC106582451 | PREDICTED: uncharacterized LOC106582451 |
| Ssa22 | ppcs | phosphopantothenoylcysteine synthetase |
| Ssa22 | LOC106582868 | PREDICTED: macrophage-stimulating protein receptor-like |
| Ssa22 | LOC106582870 | PREDICTED: metabotropic glutamate receptor 6-like |
| Ssa22 | LOC106583010 | PREDICTED: polyhomeotic-like protein 2 |
| Ssa22 | LOC106583145 | PREDICTED: collagen alpha-1(VII) chain-like |
| Ssa23 | LOC106584197 | PREDICTED: rho GTPase-activating protein 39-like |
| Ssa23 | LOC106584731 | PREDICTED: amyloid beta A4 precursor protein-binding family A member 1-like |
| Ssa24 | LOC106585065 | PREDICTED: single-stranded DNA-binding protein 2-like |
| Ssa24 | LOC106585253 | PREDICTED: myeloperoxidase-like |
| Ssa24 | LOC106585253 | PREDICTED: myeloperoxidase-like |
| Ssa25 | ube2f | ubiquitin-conjugating enzyme E2F (putative) |
| Ssa25 | rftn2 | raftlin family member 2 |
| Ssa26 | LOC106587191 | PREDICTED: ubiquitin carboxyl-terminal hydrolase 47-like |
| Ssa27 | LOC106588264 | PREDICTED: flotillin-1-like |
| Ssa27 | LOC106588408 | PREDICTED: zinc finger protein 384-like |
| Ssa27 | LOC106588403 | PREDICTED: death domain-associated protein 6-like |
| Ssa27 | tapbp | TAP binding protein |
| Ssa27 | brd2 | bromodomain containing 2 |
| Ssa27 | LOC106588261 | PREDICTED: flotillin-1-like 3-oxoacyl-[acyl-carrier-protein] reductase FabG-like |
| Ssa27 | LOC106588396 | PREDICTED: collagen alpha-2(XI) chain-like |
| Ssa27 | LOC106589083 | PREDICTED: homeobox-containing protein 1-like |
| Ssa28 | LOC106589392 | PREDICTED: ATPase family AAA domain-containing protein 1-B |
| Ssa29 | arhgap29 | Rho GTPase activating protein 29 |

| Table S2. Genes identified in Scottish populations, using Atlantic salmon annotation (GCF_000233375.1), which is a complete representation of the genome constructed by using RNA sequencing (RNA-seq) and expressed sequence tags (ESTs) that were mapped to the PFAM, stickleback and zebrafish databases (Lien et al. 2016) | | |
| --- | --- | --- |
| CHROM | **GENE** | **PRODUCT** |
| Ssa01 | LOC106569018 | PREDICTED: kinesin-like protein KIF26B |
| Ssa01 | LOC106582637 | PREDICTED: RAC-alpha serine/threonine-protein kinase-like |
| Ssa01 | LOC106587081 | PREDICTED: ryanodine receptor 3-like |
| Ssa01 | LOC106599786 | PREDICTED: exonuclease 3\'-5\' domain-containing protein 1-like |
| Ssa01 | aqr | aquarius intron-binding spliceosomal factor |
| Ssa01 | ttc6 | tetratricopeptide repeat domain 6 |
| Ssa01 | slc25a21 | solute carrier family 25 (mitochondrial oxoadipate carrier)-member 21 |
| Ssa01 | LOC106604517 | PREDICTED: myopalladin-like |
| Ssa01 | LOC106607945 | PREDICTED: attractin-like protein 1 |
| Ssa01 | LOC106613059 | PREDICTED: leucine zipper putative tumor suppressor 2 homolog |
| Ssa01 | LOC106612695 | PREDICTED: dystonin-like |
| Ssa01 | kiaa2026 | KIAA2026 ortholog |
| Ssa01 | LOC106565751 | PREDICTED: BMP/retinoic acid-inducible neural-specific protein 1-like |
| Ssa01 | LOC106565761 | uncharacterized LOC106565761 |
| Ssa03 | LOC106600700 | PREDICTED: transient receptor potential cation channel subfamily M member 4-like |
| Ssa04 | zn271 | Zinc finger protein 271 |
| Ssa04 | ryr1 | ryanodine receptor 1 (skeletal) |
| Ssa04 | LOC106603118 | PREDICTED: spectrin beta chain, non-erythrocytic 1-like |
| Ssa04 | LOC106603171 | PREDICTED: gamma-aminobutyric acid receptor subunit alpha-3-like |
| Ssa04 | LOC106603263 | PREDICTED: roundabout homolog 2-like |
| Ssa04 | LOC106603326 | PREDICTED: SH3 domain-binding glutamic acid-rich-like protein |
| Ssa04 | LOC106603328 | PREDICTED: bromodomain and WD repeat-containing protein 3-like |
| Ssa04 | LOC106603339 | PREDICTED: signal-induced proliferation-associated 1-like protein 3 |
| Ssa04 | qpctl | glutaminyl-peptide cyclotransferase-like |
| Ssa04 | LOC106603376 | PREDICTED: arf-GAP with Rho-GAP domain, ANK repeat and PH domain-containing protein 1-like |
| Ssa04 | ercc6l | excision repair cross-complementation group 6-like |
| Ssa04 | LOC106603377 | PREDICTED: long-chain-fatty-acid--CoA ligase 4-like |
| Ssa04 | LOC106603378 | PREDICTED: cohesin subunit SA-2 |
| Ssa04 | LOC106603380 | PREDICTED: glutamate receptor 3 |
| Ssa04 | LOC106603382 | PREDICTED: neuroligin-3-like |
| Ssa04 | rb1 | retinoblastoma 1 |
| Ssa04 | LOC106603604 | PREDICTED: ceramide-1-phosphate transfer protein-like |
| Ssa04 | LOC106603603 | PREDICTED: acetylcholine receptor subunit gamma-like |
| Ssa04 | LOC106603600 | PREDICTED: misshapen-like kinase 1 |
| Ssa04 | LOC100380691 | PREDICTED: guanine nucleotide-binding protein G(I)/G(S)/G(T) subunit beta-2 |
| Ssa04 | LOC106603586 | PREDICTED: E3 ubiquitin-protein ligase Siah2 |
| Ssa04 | LOC106603552 | PREDICTED: nuclear receptor-interacting protein 1-like |
| Ssa04 | LOC106603537 | PREDICTED: UV radiation resistance-associated gene protein-like |
| Ssa04 | LOC106603526 | PREDICTED: protein BTG3-like |
| Ssa04 | LOC106603511 | PREDICTED: active breakpoint cluster region-related protein-like |
| Ssa04 | LOC106603662 | PREDICTED: opioid-binding protein/cell adhesion molecule homolog |
| Ssa04 | LOC106603701 | PREDICTED: myb-related transcription factor, partner of profilin-like |
| Ssa04 | LOC106603804 | PREDICTED: disks large homolog 2 |
| Ssa05 | LOC106604630 | PREDICTED: complexin-1-like |
| Ssa06 | LOC106607674 | PREDICTED: serine/threonine-protein kinase D3-like |
| Ssa06 | LOC106607698 | PREDICTED: sorting nexin-14-like |
| Ssa09 | LOC106610698 | PREDICTED: probable E3 ubiquitin-protein ligase RNF144A-A |
| Ssa09 | zdhhc9 | zinc finger- DHHC-type containing 9 |
| Ssa09 | LOC106611554 | PREDICTED: follistatin-related protein 5-like |
| Ssa09 | LOC106611581 | PREDICTED: teneurin-3-like |
| Ssa09 | LOC106611959 | PREDICTED: AF4/FMR2 family member 2-like |
| Ssa09 | LOC106612467 | PREDICTED: high affinity cGMP-specific 3\',5\'-cyclic phosphodiesterase 9A-like |
| Ssa10 | LOC106613518 | PREDICTED: prolyl 3-hydroxylase 2-like |
| Ssa10 | LOC106613666 | PREDICTED: unconventional myosin-IXb-like |
| Ssa10 | evi5 | ecotropic viral integration site 5 |
| Ssa10 | LOC106561046 | PREDICTED: dual specificity protein phosphatase 8-like |
| Ssa11 | LOC106561944 | PREDICTED: lysosomal acid phosphatase-like |
| Ssa11 | LOC106561991 | PREDICTED: A disintegrin and metalloproteinase with thrombospondin motifs 18-like |
| Ssa11 | LOC106563159 | PREDICTED: splicing factor, suppressor of white-apricot homolog |
| Ssa12 | LOC106564415 | PREDICTED: zinc finger protein 271-like |
| Ssa12 | chchd6 | coiled-coil-helix-coiled-coil-helix domain containing 6 |
| Ssa12 | LOC106565793 | PREDICTED: nuclear receptor coactivator 3-like |
| Ssa12 | LOC106566064 | PREDICTED: guanine nucleotide-binding protein G(i) subunit alpha-2 |
| Ssa13 | qars | glutaminyl-tRNA synthetase |
| Ssa13 | sema3g | semaphorin 3G |
| Ssa13 | LOC106567958 | PREDICTED: autism susceptibility gene 2 protein-like |
| Ssa14 | tekt2 | tektin 2 (testicular) |
| Ssa14 | LOC106570016 | PREDICTED: microtubule-actin cross-linking factor 1-like |
| Ssa14 | LOC106570217 | PREDICTED: collagen alpha-1(IX) chain-like |
| Ssa14 | LOC106570216 | PREDICTED: collagen alpha-1(XXII) chain-like |
| Ssa14 | LOC106570233 | PREDICTED: collagen alpha-1(XXII) chain-like, partial |
| Ssa14 | LOC106570392 | PREDICTED: endothelin-1-like |
| Ssa14 | LOC106570503 | PREDICTED: histone acetyltransferase KAT2B-like |
| Ssa14 | LOC106570512 | PREDICTED: collagen alpha-2(IX) chain-like |
| Ssa14 | ankrd12 | ankyrin repeat domain 12 |
| Ssa15 | LOC106571069 | PREDICTED: regulator of G-protein signaling 6-like |
| Ssa16 | LOC106573250 | PREDICTED: nuclear factor of activated T-cells, cytoplasmic 3-like |
| Ssa16 | LOC106573194 | PREDICTED: protein C12orf4 homolog |
| Ssa16 | LOC106573180 | PREDICTED: transmembrane and TPR repeat-containing protein 2-like |
| Ssa16 | LOC106573397 | PREDICTED: ETS homologous factor-like |
| Ssa17 | LOC106576042 | PREDICTED: acyl-coenzyme A thioesterase 5-like |
| Ssa17 | LOC100286412 | PREDICTED: laminin subunit beta-1 |
| Ssa18 | pygb | glycogen phosphorylase B |
| Ssa18 | coda1 | Collagen alpha-1XIII chain |
| Ssa18 | LOC106577125 | PREDICTED: pro-neuregulin-3, membrane-bound isoform-like |
| Ssa18 | LOC106577493 | PREDICTED: VPS10 domain-containing receptor SorCS2-like |
| Ssa18 | LOC106577507 | PREDICTED: calcium/calmodulin-dependent protein kinase type II delta 1 chain |
| Ssa18 | LOC106577504 | PREDICTED: ankyrin-2-like |
| Ssa18 | LOC106577539 | PREDICTED: neurexin-2-like |
| Ssa19 | LOC106579195 | PREDICTED: ankyrin repeat and fibronectin type-III domain-containing protein 1-like |
| Ssa20 | LOC106580056 | PREDICTED: transient receptor potential cation channel subfamily M member 3-like |
| Ssa20 | LOC106580749 | PREDICTED: G-protein coupled receptor 4-like |
| Ssa21 | LOC106581674 | PREDICTED: poly(rC)-binding protein 3 |
| Ssa23 | LOC106583888 | PREDICTED: seizure protein 6 homolog |
| Ssa23 | LOC106584141 | PREDICTED: carboxyl-terminal PDZ ligand of neuronal nitric oxide synthase protein-like |
| Ssa24 | LOC106585164 | PREDICTED: A disintegrin and metalloproteinase with thrombospondin motifs 12-like |
| Ssa24 | LOC106585019 | PREDICTED: urea transporter 2-like |
| Ssa25 | LOC106586120 | PREDICTED: histone deacetylase 4-like |
| Ssa25 | LOC106586260 | PREDICTED: NADH-ubiquinone oxidoreductase 75 kDa subunit, mitochondrial-like |
| Ssa25 | ino80d | INO80 complex subunit D |
| Ssa25 | LOC106586093 | PREDICTED: solute carrier family 15 member 1-like |
| Ssa25 | LOC106586311 | PREDICTED: R3H domain-containing protein 1-like |
| Ssa25 | ube2f | ubiquitin-conjugating enzyme E2F (putative) |
| Ssa25 | LOC106586343 | PREDICTED: inactive phospholipase C-like protein 1, partial |
| Ssa25 | LOC106586387 | PREDICTED: diacylglycerol kinase beta-like |
| Ssa25 | LOC106586434 | PREDICTED: low-density lipoprotein receptor-related protein 1B-like |
| Ssa25 | LOC106586456 | PREDICTED: titin-like |
| Ssa25 | esd | esterase D |
| Ssa25 | LOC106586581 | PREDICTED: uncharacterized protein LOC106586581 |
| Ssa25 | ccdc93 | coiled-coil domain containing 93 |
| Ssa25 | mspd2 | Motile sperm domain-containing protein 2 |
| Ssa25 | LOC106586618 | PREDICTED: G-protein coupled receptor 143-like |
| Ssa25 | LOC106586619 | PREDICTED: F-box-like/WD repeat-containing protein TBL1X |
| Ssa25 | fhl2 | four and a half LIM domains 2 |
| Ssa26 | LOC106587235 | PREDICTED: secretory carrier-associated membrane protein 5-like |
| Ssa27 | vps52 | vacuolar protein sorting 52 homolog (S. cerevisiae) |
| Ssa27 | LOC106588341 | PREDICTED: protein FAM63A-like |
| Ssa27 | LOC106588610 | PREDICTED: brachyury protein homolog A |
| Ssa27 | LOC106588644 | PREDICTED: zona pellucida sperm-binding protein 3-like |
| Ssa27 | slc25a13 | solute carrier family 25 (aspartate/glutamate carrier) member 13 |
| Ssa28 | LOC106589617 | PREDICTED: protein tweety homolog 2-like |

| **Table S3.** Results of Bayescan analysis in Sct-D/Sct-W populations, with no MAF and HWE filters. In Can-D/Can-W this analysis revealed none loci putatively selected. | | | | | |
| --- | --- | --- | --- | --- | --- |
| **Sct-D/Sct-W** | | | | | |
| Chromosome | Initial SNPs number | Strong | Very Strong | Decisive | **Total** |
| Ssa01 | 12041 | 5 | 4 | 6 | **15** |
| Ssa02 | 3799 |  |  |  |  |
| Ssa03 | 6229 | 2 |  |  | **2** |
| Ssa04 | 4744 |  | 1 | 6 | **7** |
| Ssa05 | 6035 | 1 |  |  | **1** |
| Ssa06 | 5291 |  |  |  |  |
| Ssa07 | 4311 |  |  |  |  |
| Ssa08 | 1451 |  |  |  |  |
| Ssa09 | 9357 | 1 | 1 | 1 | **3** |
| Ssa10 | 8194 | 1 | 2 | 3 | **6** |
| Ssa11 | 6155 | 1 | 2 | 2 | **5** |
| Ssa12 | 6232 |  |  | 2 | **2** |
| Ssa13 | 7055 |  | 1 | 1 | **2** |
| Ssa14 | 6500 | 2 |  | 2 | **4** |
| Ssa15 | 7184 |  |  | 1 | **1** |
| Ssa16 | 5405 |  | 1 | 3 | **4** |
| Ssa17 | 3262 |  | 3 |  | **3** |
| Ssa18 | 4847 |  | 4 | 3 | **7** |
| Ssa19 | 5556 |  |  |  |  |
| Ssa20 | 5965 |  | 2 |  | **2** |
| Ssa21 | 3248 | 1 |  | 3 | **4** |
| Ssa22 | 4464 |  |  |  |  |
| Ssa23 | 3458 |  |  | 2 | **2** |
| Ssa24 | 3395 |  |  | 1 | **1** |
| Ssa25 | 3759 | 9 | 6 | 9 | **24** |
| Ssa26 | 2315 |  |  |  |  |
| Ssa27 | 3239 |  | 1 | 1 | **2** |
| Ssa28 | 2383 |  | 1 | 1 | **2** |
| Ssa29 | 3333 |  |  | 1 | **1** |
| Unknown | 2302 | 2 | 1 | 1 | **4** |
| **Total** | **151509** | **25** | **30** | **49** | **104** |
